# SANS investigation of fungal loosenins reveal substrate dependent impacts of protein 1 action on inter-fibril distance and packing order of cellulosic substrates

**DOI:** 10.21203/rs.3.rs-4769386/v1

**Published:** 2024-08-14

**Authors:** Deepika Dahiya, Zsuzsanna Péter-Szabó, Manjula Senanayake, Sai Venkatesh Pingali, Wellington C. Leite, James Byrnes, Garry W. Buchko, Pramod Sivan, Francisco Vilaplana, Emma Master, Hugh O'Neill

## Abstract

Background: Microbial expansin-related proteins include fungal loosenins, which have been previously shown to disrupt cellulose networks and enhance the enzymatic conversion of cellulosic substrates. Despite showing beneficial impacts to cellulose processing, detailed characterization of cellulosic materials after loosenin treatment is lacking. In this study, small-angle neutron scattering (SANS) was used to investigate the effects of three recombinantly produced loosenins that originate from Phanerochaete carnosa, PcaLOOL7, PcaLOOL9, and PcaLOOL12, on the organization of holocellulose preparations from Eucalyptus and Spruce wood samples. Results: Whereas the SANS analysis of Spruce holocellulose revealed an increase in interfibril spacing of neighboring cellulose microfibrils following treatment with PcaLOOL12 and to a lesser extent PcaLOOL7, the analysis of Eucalyptus holocellulose revealed a reduction in packing number following treatment with PcaLOOL12 and to a lesser extent PcaLOOL9. Parallel SEC-SAXS characterization of PcaLOOL7, PcaLOOL9, and PcaLOOL12 indicated the proteins likely function as monomers; moreover, all appear to retain a flexible disordered N-terminus and folded C-terminal region. The comparatively high impact of PcaLOOL12 motivated its NMR structural characterization, revealing a double-psi b-barrel (DPBB) domain surrounded by three alpha-helices - the largest nestled against the DPBB core and the other two part of loops extending from the core. Conclusions: The SANS analysis of PcaLOOL action on holocellulose samples confirms their ability to disrupt cellulose fiber networks and suggests a progression from reducing microfibril packing to increasing interfibril distance. The most impactful PcaLOOL, PcaLOOL12, was previously observed to be the most highly expressed loosenin in P. carnosa. Its structural characterization herein reveals its stabilization through two disulfide linkages, and an extended N-terminal region distal to a negatively charged and surface accessible polysaccharide binding groove.

### BACKGROUND

Expansins are an intriguing group of plant proteins that induce plant cell loosening through promoting cell wall creep and stress relaxation at acidic pH (1–3). Although the molecular mechanism of expansins is not fully understood, all expansins characterized to date lack detectable lytic activity. Instead, these proteins are thought to physically disrupt non-covalent interactions between cellulose and matrix polysaccharides (e.g., pectin, hemicelluloses), potentially at distinctive and limited locations characterized by tight junctions between neighboring cellulose microfibrils (4,5).

Over the past decade, genome sequencing has uncovered the prevalence of expansin-like proteins in microorganisms, including bacteria and fungi (6–9). Both plant expansins and microbial expansin-like proteins (EXLX) exhibit a two-domain structure comprising an N-terminal six-stranded double-*psi* b-barrel domain (D1) resembling glycoside hydrolases from family 45 (GH45) although lacking all requisite catalytic amino acids. The D1 domain is connected through a short linker to a C-terminal domain (D2) that adopts a fold similar to group 2 grass pollen allergens and is classified as a family-63 carbohydrate binding module (CBM63) (10). The comparative ease to recombinantly produce microbial EXLX proteins, such as *Bs*EXLX1 from *Bacillus subtilis*, has advanced sequence-functional characterizations of the expansin protein family (10–12). For example, structural and mutagenesis studies of *Bs*EXLX1 confirm the critical importance of Asp82 in the D1 domain to protein function, as well as the importance of aromatic amino acids in the D2 domain, including Trp125 and Trp126, to cellulose binding (11).

Besides EXLX proteins, the broader set of microbial expansin-related proteins include loosenins and ceratoplatanins that retain only the D1 domain of EXLXs, and swollenins that comprise an N-terminal family-1 CBM and fibronectin domain in addition to the core EXLX structure (13-15). Notably, the swollenin *Tr*SWO1 from *Trichoderma reesei* exhibits low hydrolytic activity towards soluble cello-oligosaccharides (16), which might be explained by the several insertions and deletions in the D1 domain that differentiate swollenins from other expansin-related proteins (17).

So far, most studies of microbial expansin-related proteins have investigated their potential to boost enzymatic hydrolysis of lignocellulosic substrates (17–21). Overall, the impact of microbial expansin-related proteins on the enzymatic deconstruction of lignocellulose varies and depends on the lignocellulose source, enzyme cocktail, and enzyme dose (22,23). Different biophysical methods have also been used to study the impact of microbial expansins on the structure of cellulose fibers. For example, studies using light microscopy show treatment of mercerized cotton fiber with *Tr*SWO1 induces fiber swelling (15). On the other hand, subsequent studies using scanning electron microscopy and atomic force microscopy show that treatment of cellulosic material with *Tr*SWO1 leads to smoother fiber surfaces (24). Nevertheless, *Tr*SWO1 reduces the tensile strength of filter paper (15) as has been similarly reported for several microbial expansin-like proteins such as *Bs*EXLX1(11), *Hc*EXLX2 from *Hahella chejuensis* (25), *Pc*Exl1 from *Pectobacterium carotovorum* (26), and loosenins including LOOS1 from *Bjerkandera adusta* (14) and *Pca*LOOLs from *Phanerochaete carnosa* (27). Clearly, additional and direct comparisons of multiple expansin-related proteins on defined lignocellulosic substrates are needed to elucidate their mode of action and substantiate sequence and substrate dependent impacts on protein function.

Small-angle neutron scattering (SANS) is a powerful approach to characterizing the morphology and organization of a wide variety of complex materials (28). In particular, it has been used to characterize plant cell wall structure under a variety of conditions providing nanoscale information about cell wall organization. For instance, it can simultaneously measure changes in intra-fibrillar (e.g., swelling) and inter-fibrillar (e.g., organization) cellulose fiber structures (29). Moreover, because SANS is a non-destructive analysis method, biological samples can be recovered for parallel investigations. SANS was previously used to investigate the impact of cellulolytic enzymes and chelator-mediated Fenton (CMF) chemistry on southern yellow pine and revealed an increase in inter-fibril spacing following CMF treatment that was enhanced when treating delignified wood fiber (30). Here, SANS is used to compare the action of three loosenins from *P. carnosa* (*Pca*LOOL7, *Pca*LOOL9, and *Pca*LOOL12) on wood holocellulose fibers, i.e. cellulosic fibers that have been partially delignified but retain most of the hemicellulose populations, from two representative wood sources (Spruce softwoods and Eucalyptus hardwoods) with distinct lignin and hemicellulose compositions. The selected *Pca*LOOLs were previously shown to weaken filter paper, reduce yield strain of cellulose nanofibers, and improve cellulolytic conversion of complex lignocellulosic substrates including steam pretreated softwood and hardwood and softwood kraft pulps (21,27). The current SANS analyses of these proteins deepen our understanding of their mode of action, thereby shedding light on the biological relevance of this protein family and their application in biomass processing.

**METHODS**

**Materials**. Wood chips from softwood Spruce *Picea abies* and hardwood *Eucalyptus grandis* were provided by UPM-Kymene Oyj (Helsinki, Finland) and were used to prepare substrate specimens for protein treatment. The wood chips were cut to a matchstick size (approximately 2 mm in the tangential x 1 mm in radial x 20 mm in the longitudinal direction of the wood section). Before delignification, low molecular weight wood extractives and resins were removed according to the SCAN-CM 49:03 standard procedure. Acetone Soxhlet extraction was carried out for 6 h, during which time the spent solvent was replaced with fresh solvent every hour.

**Holocellulose preparation and characterization.** Acetone extracted wood sections (1 g) were mixed in 15 mL deionized water using magnetic stirrer and heated to 75 °C. Glacial acetic acid (0.1 mL) and 0.3 g $NaClO_2$ per g of wood section were then added every hour for 6 h (31,32).

For total carbohydrate analysis, freeze dried native and holocellulose wood samples were incubated with concentrated 72 w/w% sulfuric acid at 8 mg/mL for 3 h at room temperature. The samples were then diluted to 0.67 mg/mL with Milli-Q water and hydrolyzed at 100 °C for 3 h (33). Parallel treatments were performed to quantify both the neutral monosaccharides and uronic acids of the non-cellulosic polysaccharides in the wood and the holocellulose samples by trifluoracetic acid (TFA) hydrolysis. Briefly, 1 mg of the solid samples were hydrolyzed at 1 mg/mL in 2 M trifluoracetic acid (TFA) for 3 h at 120 °C, dried under airflow, and dissolved in 1 mL Milli-Q water. The resulting monosaccharides from both sulfuric and TFA hydrolyses were then quantified using high-performance anion-exchange chromatography coupled with pulsed amperometric detection (HPAEC-PAD)(34). The HPAEC-PAD analysis was performed using a Dionex ICS-6000 system (Thermo Fischer, MA, USA) equipped with a CarboPac PA20 (3 × 150 mm) column. The eluents for chromatography were Milli-Q water (eluent A), 200 mM NaOH (eluent B), and 100 mM sodium acetate in 100 mM NaOH (eluent C) at a flow rate of 0.4 mL/min. The neutral sugars were separated over 18 min in 98.8 % eluent A and 1.2 % eluent B (2.4 mM NaOH). The column was then equilibrated with 50 % eluent A and 50 % eluent B for 10 min, and the uronic acids were further eluted over 16 min at 100 % eluent C. The quantification of each monosaccharide was performed using calibration curves of neutral sugars and uronic acid standards (fucose, arabinose, rhamnose, glucose, xylose, mannose, galacturonic acid, glucuronic acid, and 4-*O*-methyl-glucuronic acid).

The total lignin content of the initial wood samples was determined using the acetyl bromide method (35,36) with modifications to prevent xylan degradation, which can interfere with lignin quantification (37). Briefly, 5 mg of freeze-dried and ball milled wood powder was weighed and digested with 1 mL 25 v/v % acetyl bromide in glacial acetic acid solution for 2 h at 50 °C. Samples were cooled on ice and diluted with 5 mL glacial acetic acid; 90 µL of the diluted sample were then mixed with 120 µL of 2 M NaOH and 90 µL 0.5 M hydroxylamine hydrochloride solution to achieve a 3:4:3 volumetric ratio of sample: 2M NaOH: 0.5M hydroxylamine hydrochloride. Absorbance was measured at 280 nm using a BMG Labtech CLARIOstar spectrophotometer (BMG Labtech, Ortenberg, Germany). Calculations of lignin concentration were performed using a calibration curve of acetyl bromide digested alkali lignin.

**Recombinant production and purification.** Three loosenins from *Phanerochaete carnosa* (*Pca*LOOL7 (GenBank code EKM53490.1), *Pca*LOOL9 (GenBank code EKM52742.1) and *Pca*LOOL12 (GenBank code EKM51974.1) were recombinantly produced with a C-terminal $His_6$-tag in *Pichia pastoris* SMD1168H according to the Pichia fermentation guidelines and using a 7 L Sterilizable-In-Place (SIP) Fermenter (BIOSTAT® Cplus bioreactor, Sartorius, Göttingen, Germany) fitted with pH (Article no. BB-34090812), oxygen (Article no. BB-8848663) and turbidity HAMILTON probes from Sartorius (21, 27). Precultures for the bioreactor were prepared by transferring fresh colonies of each transformant from YPD agar plates (supplemented with zeocin) into 300 mL BMGY (Buffered Glycerol-complex Medium) and growing the cultures at 30 °C and 180 rpm for 16-24 h until the $OD_{600nm}$ reached between 20-25. The pre-culture medium was replaced with 50 mL of sterile minimal basal salts medium (BSM) before transferring the cells to the bioreactor containing sterilized 3.5 L BSM media containing 4 % (w/v) glycerol and 0.4 % (v/v) PTM1 fermentation trace salts (VWR, PA, USA). The reactor was supplemented with approximately 100 µL of antifoaming agent Struktol J 647 to prevent the formation of foam throughout the fermentation. The glycerol growth phase was performed at 30 °C and pH 6.0. The maximum stirring rate was set to 900 rpm, air flow rate to 40-60 %, volume of air per volume of liquid per minute (VVM) between 0.5-1.0, and the oxygen level was set to 35 %. The oxygen was maintained by using automatic cascade with stirring and gas flow until the cells approached the stationary phase, at which time the Glycerol Fed-Batch Phase was initiated with up to 4 % w/v of glycerol feeding for over 5 to 6 h to achieve higher cell density. To induce recombinant protein production, the temperature was lowered to 20 °C and methanol was added at up to 6.5 mL/ h/ L of media for up to 110 h.

Following induction, the pH of the culture was increased to pH 7.8 using 4 M NaOH before removing the cells by centrifugation and filtering the culture supernatant through a 0.45 µm polyethersulfone (PES) filter (Millipore). The recombinant *Pca*LOOLs were then purified by affinity chromatograph using Ni-NTA resin as previously described (27). The purified protein was exchanged to 20 mM sodium acetate (pH 6.0) and concentrated to 10-15 mg/mL using Vivaspin® 20 Ultrafiltration Units (5 kDa and 10 kDa) from Sartorius before flash-freezing and storage at -80 °C. The protein purity was verified by SDS-PAGE and protein concentration was determined by absorbance at 280 nm using a Thermofischer Scientific NanoDrop™ Lite Spectrophotometer (Thermo, Fischer, MA, USA).

To prepare recombinant protein for NMR studies, an oligonucleotide sequence for *Pca*LOOL12, with the predicted 19-residue N-terminal signal sequence replaced with a polyhistidine affinity tag (bold) and a TEV protease cleave site (underlined), (MGSS**HHHHHH**SSGENLYFQGH-), was synthesized by Genscript (Piscataway, NJ, USA) and inserted into the pET-32a(+) expression vector at the MscI/HindIII restriction enzyme sites such that the expressed protein was fused to thioredoxin (109-residues) at the N-terminal (Trx-*Pca*LOOL12). Fusion of *Pca*LOOL12 to thioredoxin moiety was necessary to improve the expression of soluble *Pca*LOOL12 (no soluble expression observed in constructs without the thioredoxin tag) (38, 39). This recombinant plasmid was then used to transform chemically competent *Escherichia coli* BL21(DE3) cells (Novagen, Darmstadt, Germany) using a heat-shock method. Single colonies from streaked LB-agar plates supplemented with the antibiotic ampicillin (100 mg/mL) were picked, grown to an $OD_{600}$ of ~ 0.8 in 5 to 7 mL of LB medium at 37 °C and used to prepare glycerol frozen stocks (-80 °C) until required. To prepare uniformly $^{15}$N-, $^{13}$C-labeled Trx-

*Pca*LOOL12, 750 mL of minimal medium (Miller) containing $^{15}NH_4Cl$ (1 mg/mL), D-[$^{13}C_6$]glucose (2.0 mg/mL), NaCl (50 mg/mL), $MgSO_4$ (120 mg/mL), $CaCl_2$ (11 mg/mL), and ampicillin (100 mg/mL) was inoculated directly with 20 mL of LB culture that had grown at 37 °C to an $OD_{600}$ of ~ 1. The resulting culture was grown at 37 °C to an $OD_{600}$ ~ 0.8 and then transferred to a 20 °C incubator shaker and protein expression induced overnight with isopropyl β-D-1-thiogalactopyranoside (0.026 mg/mL). Approximately 12 h later the cells were harvested by mild centrifugation and frozen (-80 °C). To make stereospecific assignments of the leucine and valine methyl groups, a 10% $^{13}C$-labeled sample was prepared as described above by using D-[$^{13}C_6$]glucose (0.2 mg/mL) and D-glucose (1.8 mg/mL). Following the thawing of a frozen pellet from a 750 mL culture, the cells were lysed using a sonication and French press combination, and the soluble protein was purified with a conventional two-step protocol involving metal chelate affinity chromatography using Ni-NTA resin (GE Healthcare, Piscataway, NJ, USA) followed by gel-filtration chromatography on a Superdex75 HiLoad 26/60 column (GE Healthcare, Piscataway, NJ, USA). The latter step exchanged the protein into NMR buffer: 100 mM NaCl, 20 mM Tris, pH 7.0. Following concentration to 1-2 mL, an equal volume of TEV protease buffer (150 mM NaCl, 50 mM TrisHCl, pH 7.8) was added plus 1 μg TEV protease per 100 μg of Trx-*Pca*LOOL12. Following overnight incubation at 4 °C, the digested protein was purified by reapplication to a 5 mL Ni-NTA column. The flow-through and 10 mM imidazole fractions were collected, concentrated, and exchanged into NMR buffer to a final concentration of 10 – 15 mg/mL. Note that during the purification process none of the buffers contained reducing agents such as dithiothreitol.

**NMR data collection for *Pca*LOOL12.** The NMR data for oxidized *Pca*LOOL12 was collected at 20 °C on a double-labeled ($^{13}$C-, $^{15}$N-) sample (~ 0.8 mM) using a Varian spectrometer operating at a $^{1}$H resonance frequency of 600 MHz equipped with an HCN-cyroprobe and pulse field gradients. Backbone and side chain chemical shifts ($^{1}$H, $^{13}$C, and $^{15}$N) were assigned from the analysis of two-dimensional $^{1}$H-$^{15}$N HSQC, $^{1}$H-$^{13}$C HSQC, HBCBCGCDHD, and HBCBCGCDCHE spectra and three-dimensional HNCACB, CBCA(CO)NH, HNCA, CC-TOCSY-NNH, HNCO, $^{15}$N-edited NOESY-HSQC, and $^{13}$C-edited NOESY-HSQC (aliphatic and aromatic) spectra. The NOE data was collected with a mixing time of 90 ms. Backbone hydrogen bonds were made by lyophilizing an $^{15}$N-labeled NMR sample, re-dissolving in a similar volume of 99.8% $D_2O$, collecting a $^{1}$H-$^{15}$N HSQC spectrum (~ 10 min later), and identifying amide resonances that had not yet exchanged with $D_2O$. The overall rotational correlation time, $\tau_c$, was estimated for oxidized *Pca*LOOL12 at 20 °C from the ratio of collective backbone amide $^{15}$N $T_1$ and $T_{1rho}$ measurements (40). The raw NMR data were processed with NMRpipe (41) and then analyzed with Poky (42). The $^{1}$H, $^{13}$C, and $^{15}$N chemical shifts were deposited into the BioMagResBank database (www.bmrb.wisc.edu) with the BMRB number 31180.

**Structure calculations for *Pca*LOOL12.** Assigned $^{1}$H, $^{13}$C, and $^{15}$N chemical shifts, peak-picked $^{1}$H-$^{1}$H NOEs from $^{13}$C- and $^{15}$N-editted 3D NOESY data sets, and TALOS-derived backbone torsion angles served as initial experimental inputs in iterative structure calculations using the program CYANA (v 2.1) (43). Seventy-five dihedral Psi ($\Psi$) and Phi ($\Phi$) torsion angle restraints were obtained by the input of the assigned chemical shifts into the program TALOS+ using the online webserver (https://spin.niddk.nih.gov/bax/nmrserver/talos/) (44). Sixty-eight backbone amide hydrogen bond restraints to backbone carbonyls groups (1.8 – 2.0 Å and 2.7 – 3.0 Å for the NH–O and N-O distances, respectively) were introduced into the structure calculation based on consistent proximity in the ensemble and the observation of slowly exchanging amides in the deuterium exchange experiment. Six restraints between the side chain sulfur atoms of C26-C50, and C73-C76 (2.0-2.1 Å, 3.0-3.1 Å, and 3.0-3.1 Å for the Sγ-Sγ, Sγ-Cβ, and Cβ-Sγ distances, respectively) were introduced into the calculation because of side chain proximity in early structure calculations and cysteine $^{13}C^{\beta}$ chemical shifts characteristic of oxidized thiols (Table S1) (45). Towards the end of the calculations, stereo assignments for six leucine and eight valine methyl groups were introduced. The final ensemble of CYANA structures was refined in explicit water (46) using the PARAM19 force-field and force constants of 500, 500, and 1000 kcal for the NOE, hydrogen bond, and dihedral restraints, respectively. For these calculations the upper boundary of all the final CYANA NOE-based distance restraints was increased by 10%. Structural quality was assessed using the online Protein Structure Validation Suite (PSVS, v1.5) (47) with pertinent values included in the structure statistics summary (Table S2). The atomic coordinates for the final ensemble of 20 *Pca*LOOL12 structures have been deposited in the Research Collaboratory for Structural Bioinformatics (RCSB) with the PDB ID code 9CE9.

**Size exclusion chromatography coupled to SAXS of protein samples.** Size exclusion chromatography coupled with small-angle X-ray scattering (SEC-SAXS) was performed at the Life Sciences X-ray Scattering Beamline (LiX) at NSLSII, Brookhaven National Laboratory. Briefly, 60 µL of sample (13-23 mg/mL *Pca*LOOL produced in *P. pastoris*) was injected onto a Superdex 200 Increase 5/150GL column (Cytiva) using an Agilent 1260 Infinity II Bio Inert

HPLC system (Agilent Technologies, CA, USA). The flow rate was set to 0.35 mL/min and eluate from the column was split 2:1 via a passive splitter between the X-ray flow cell and UV branch, respectively (48). SAXS/WAXS images of 2 s exposures and 350 frames were collected simultaneously on a Pilatus 1M (SAXS) and Pilatus 900K (WAXS) detector. Data from both detectors were merged, scaled, and normalized to the water peak scattering intensity using LiX beamline software, py4xs and lixtools, as described elsewhere (49,50).

**SAXS analysis of protein samples.** BioXTAS RAW program and ATSAS suite were used for analysis of the SAXS data (51,52). The pair distance distribution function ($P(r)$), implemented in RAW, was calculated using the indirect Fourier transform method using GNOM (53). The SAXS-derived molecular weight was determined using the volume of Porod method in RAW (54). The experimental SAXS data were compared to theoretical scattering curves generated from atomistic models using the FOX program (55,56). Protein conformational sampling was performed using AlphaFold2 (57) and Normal Mode Analysis (ATSAS). Chimera was used to visualize PDB structures (58).

**Treatment of native wood and holocellulose samples with *Pca*LOOLs.** Acetone extracted native wood and holocellulose samples (2 mm x 1 mm x 20 mm) were soaked in a freshly prepared sodium acetate buffer (20 mM, pH 5.5, in 100 % $D_2O$) followed by three solvent exchanges over 24 h to ensure the maximum number of accessible exchangeable hydrogen atoms were exchanged with deuterium atoms in the samples. After deuterium exchange, 1.4 mg of *Pca*LOOL proteins produced in *P. pastoris* were separately mixed with 70 mg of wood sample or holocellulose in a glass vial and the final volume adjusted to 4.0 mL with the sodium acetate buffer. Control samples were prepared in the same manner but without the addition of protein. The control and analytical samples were agitated at 400 rpm in a mechanical shaker at 40 °C for 24 h. Native, unextracted wood samples were also exchanged into $D_2O$ buffer and incubated at room temperature (~25 °C) with no shaking.

**SANS data collection.** Approximately 3-5 slices of each treated wood and holocellulose sample were carefully placed side-by-side in titanium sample cells with each slice vertically aligned perpendicular to the incident beam. The cells were filled with the deuterated sodium acetate buffer solution. SANS measurements were performed at the Bio-SANS (CG-3) instrument located at the High Flux Isotope Reactor (HFIR) in the Oak Ridge National Laboratory (ORNL). The main detector array was at 15.5 m from the sample, and the curved wing detector array at 1.13 m from the sample and rotated 1.4° from the direct beam. Using this detector configuration, the Q ranges obtained using 6 Å neutrons were $0.003 < Q$ ($Å^{-1}$) $< 0.8$, and the spread in wavelength ($\Delta\lambda/\lambda$) was 13.2%.

The vertical arrangement of the sliced wood stems maintained the inherent alignment of the plant cell wall structure, mainly cellulose microfibrils aligned along the stem growth direction. These samples produce an anisotropic 2D scattering pattern. The raw 2D images were processed by correcting for detector dark current, pixel sensitivity, normalizing to the incident beam monitor counts, and subtracting scattering contribution from quartz cell and deuterated buffer. Two scattering intensity profiles, I(Q) versus Q, were obtained from the equatorial (wedge 0) and meridional (wedge 1) sectors of the 2D image. From the different scattering contributions in the two sectors, the dominant scattering feature of the equatorial sector is the cellulose microfibril structure while in the meridional sector it is the isotropic matrix copolymer. To isolate the cellulose microfibril feature, the meridional scattering contribution was then subtracted from the equatorial sector and analyzed as detailed below. More information about wedge reduction can be found in a prior publication (59).

**SANS data analysis.** The SANS data were analyzed using the Modeling II tool implemented in the Irena package (60) in Igor Pro 8.0 software (WaveMetrics, OR, USA). The basic small-angle scattering formula for scattered intensity $I(Q)$ is

$$I(Q) = |\Delta\rho|^2 S(Q) \int_0^\infty |F(Q,r)|^2 \, V(r)^2 N P(r) dr \qquad (1)$$

where $\Delta\rho$ is the contrast between scattering particle and solvent $\left|\rho_{particle} - \rho_{solvent}\right|$, $F(Q,r)$ is the scattering form factor of the cylindrical particles, $V(r)$ is the particle volume of size $r$, $N$ is the total number of scattering particles, $P(r)$ is the Gaussian probability density distribution of the scattering particles of size $r$, and $S(Q)$ is the structure factor modeled with a hard sphere structure factor unless there is no need then $S(Q) = 1.0$. The equatorial SANS profiles of Eucalyptus and Spruce were modeled using a cylindrical form factor in the high Q region (Q ~ 0.05 – 0.4 Å$^{-1}$) for all samples to represent the cellulose microfibril. The mathematical formulation of the cylindrical form factor is given by,

$$P(Q) = \frac{scale}{V_{cyl}} \int_o^{\frac{\pi}{2}} f^2 \, (Q,\alpha) sin\alpha d\alpha \qquad (2)$$

$$f(Q,\alpha) = 2\left(\rho_{cyl} - \rho_{solv}\right) V_{cyl} j_0(Q \times Hcos\alpha) \frac{J_1(Q \times rsin\alpha)}{(Q \times rsin\alpha)} \qquad (3)$$

$$S(Q) = \frac{1}{1 + k \times \frac{3 \times (\sin(Q\zeta) - Q\zeta \times \cos(Q\zeta))}{(Q\zeta)^3}} \qquad (4)$$

where H is half the cylinder length (L=2H), $r$ is the cross-sectional radius of the cylinder, $\alpha$ is the orientation angle of the cylinder's long axis, and $J_1$ is a first-order Bessel function. The low Q data (Q ~ 0.003 – 0.05 Å$^{-1}$) was fit to a power-law function (using the Unified Fit function), given by $I(Q) = B * Q^{-\alpha}$, where $\alpha$ is the power-law slope of the scattering curves, and $B$ is the scale factors of the power-law function (61). For subtracted equatorial 1D SANS profiles of samples and controls, a structure factor was also included in the fitting. The convergence of the fit using Genetic optimization was based on minimizing the chi-squared ($\chi^2$) value of the fit for the entire data range. The structure factor that accounts for the weak correlation is given in equation 4 and is used to model the correlation between cellulose microfibrils. Here, $k$ describes the degree of correlation ($0<k<5.92$), and $\zeta$ is the average center-to-center distance between microfibrils (62).

The meridional SANS data of Eucalyptus and Spruce were modelled by using a three-level fit. The high Q (Q ~ 0.075 - 0.4 Å$^{-1}$) region was modelled by the spherical form factor given as,

$$P(q) = \frac{\phi}{V_{sph}} \frac{3\left[\sin(QR_{sph}) - QR_{sph}cos(QR_{sph})\right]^2}{\left(QR_{sph}\right)^3} \qquad (5)$$

where, $F$ is the volume fraction of spheres and $V_{sph} = \frac{4}{3}\pi R_{sph}^3$ is the volume of a sphere of radius $R_{sph}$. In addition, two slopes of different Q-exponents were observed in the intermediate Q region (Q ~ 0.01 – 0.075 Å$^{-1}$) and low Q region (Q ~ 0.003 – 0.01 Å$^{-1}$), and these two regions were fit using two power-law functions (by using two levels of the Unified Fit function) described above (60).

### RESULTS AND DISCUSSION

Three loosenins from *P. carnosa* (*Pca*LOOL7, *Pca*LOOL9, and *Pca*LOOL12) were characterized in terms of their solution structures and impacts on lignocellulosic materials. For these studies, *Pca*LOOL7, *Pca*LOOL9 and *Pca*LOOL12 were recombinantly produced in *P. pastoris*, achieving yields of 174, 479, and 303 mg/L, respectively (Figure S1). SANS was then used to compare the structural effects of these loosenins on holocellulose substrates prepared from softwood (Spruce, *Picea abies*) and hardwood (Eucalyptus, *Eucalyptus grandis*) chips that comprise varying lignin and hemicellulose contents.

**Structural characterization of the *Pca*LOOLs used for holocellulose treatment.** Besides recombinant production in *P. pastoris*, it was possible to obtain milligram amounts of soluble *Pca*LOOL12 in *E. coli* by fusing thioredoxin to the N-terminal of the protein. *Pca*LOOL12 produced in *E. coli* remained soluble following thioredoxin cleavage and removal, enabling the $^{13}C$- and $^{15}N$-labeling of *Pca*LOOL12 and calculation of an NMR solution structure. The assigned $^{1}H$-$^{15}N$ HSQC spectrum for *Pca*LOOL12, illustrated in Figure S2, features sharp and uniform cross peaks with wide chemical shift dispersions in both dimensions, general characteristics of a small, folded protein (63). The estimated rotational correlation time for *Pca*LOOL12, calculated from a series of 1-D, $^{15}N$-edited proton spectra, was $4.7 \pm 1.1$ ns, a value expected for a 10.8 kDa protein (64). These features, along with a late elution time on a Superdex75 size exclusion column (data not shown), indicated that *Pca*LOOL12 was a monomer in solution.

As illustrated in Figure S2 and tabulated in the chemical shift assignments deposited into the BMRB, 96 out of the expected 99 backbone amide resonance cross peaks (105 – (N-terminal + 5

prolines)) were assigned in the $^{1}H$ -$^{15}N$ HSQC spectrum of *Pca*LOOL12. This enabled nearly complete assignment of all the backbone and side chain protons critical for the auto-assignment of the peak-picked NOE data. Consequently, as summarized in Table S2, 1300 NOE based distance restraints (of which nearly 500 were long range), 68 hydrogen bond restraints, a total of 150 Phi and Psi torsion angle restraints, and 6 disulfide bond restraints were used in the final set of structure calculations. This resulted in good convergence of the final ensemble of 20 structures as shown in the cartoon ensemble, superimposed over the ordered regions, in Figure 1B. The RMSD of the structured core from the mean structure was 0.50 ± 0.07 Å for the backbone atoms (N-$C^{\alpha}$-C=O) and 0.84 ± 0.06 Å for all heavy atoms (Table S2). The good quality of the final set of structures is further corroborated by an analysis of these structures with the PSVS validation software package (47) as tabulated in Table S2. Ramachandran statistics for the Phi/Psi pairs were all in favored (90.7 %) or additionally allowed (9.3 %) regions with the structure-quality Z-scores all above acceptable levels (>-4).

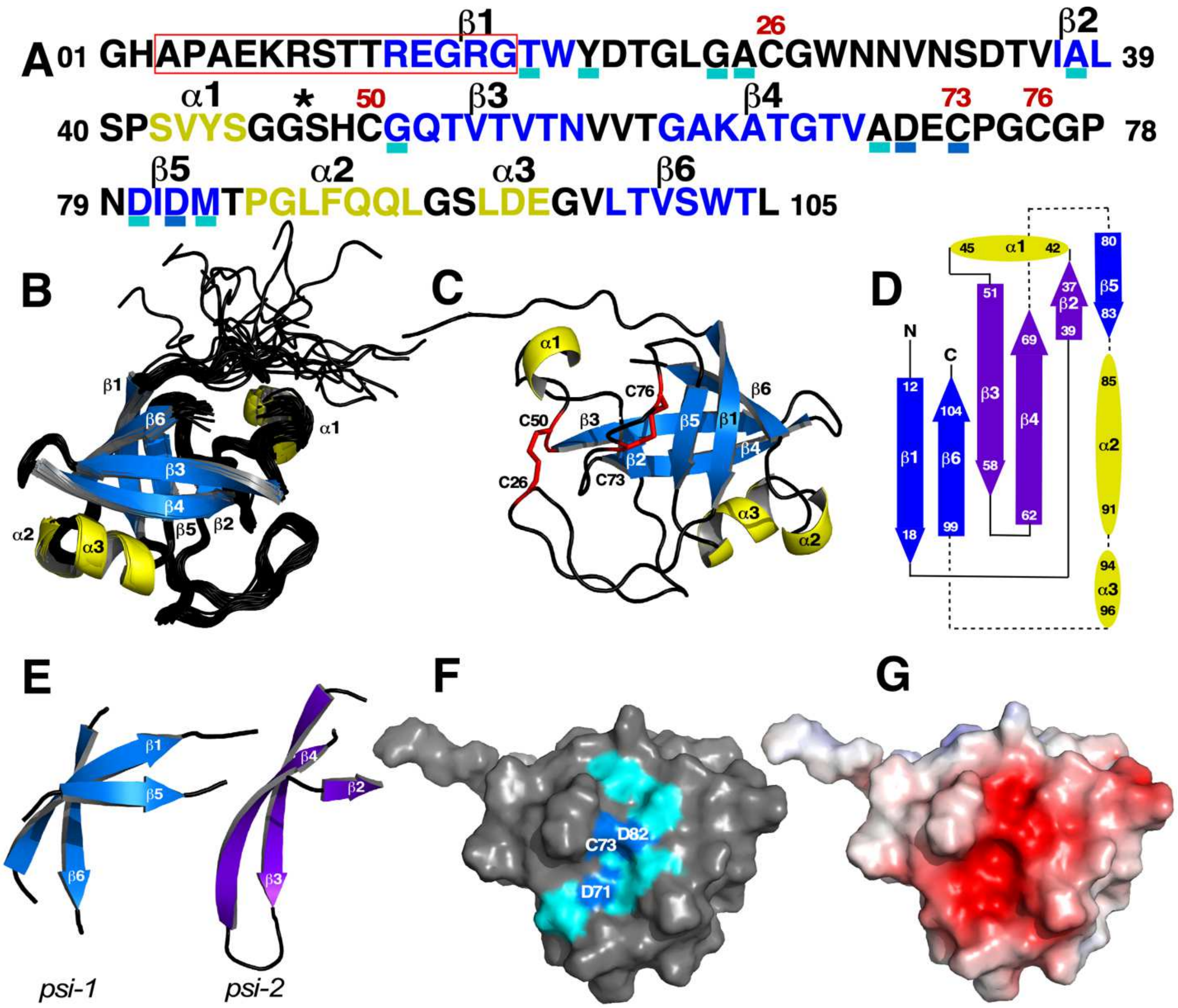

**Figure 1.** Solution NMR structure of *Pca*LOOL12 (9CE9). (A) The primary amino acid sequence of *Pca*LOOL12 with the β-strand and α-helix elements of secondary structure colored blue and yellow, respectively. Disulfide bonds are observed between C26-C50 and C73-C76 with the position of these four cysteine residues identified with red residue numbers above the sequence. The *P. carnosa* genome contains 12 loosenin genes divided into two subgroups based on the position of an insertion relative to the predicted polysaccharide binding groove (*Pca*LOOL12 belongs in subgroup B) (15). The red box is the characteristic 12-18 residue insertion observed in subgroup B loosenins and the asterisk after G44 indicates the position of the characteristic 8-9 residue insertion observed in subgroup A loosenins. The rectangles below the sequence denote conserved residues in the *P. carnosa* loosenin family predicted to form a polysaccharide binding groove, with the blue rectangles denoting residues associated with expansin activity (EXLX1) in this groove. The first two N-terminal residues are “scars” left over after cleavage of the thioredoxin moiety of the original construct. The six $\beta$-strands form a double-*psi* $\beta$-barrel (DPBB) structural fold. (B). A cartoon superposition of the ensemble of 20 solution structures calculated for *Pca*LOOL12. (C) A cartoon representation of a single structure in the ensemble rotated 180 ° in the y-axis to better illustrate the location of the two disulfide bonds (side chains colored red) and the three $\beta$-strands on the back side of the protein. (D) A topology diagram illustrating the relative orientation of the elements of secondary structure with the $\alpha$-helices colored yellow and the $\beta$-strands colored blue (*psi-1*) or purple (*psi-2*). The $\beta$-strands and $\alpha$-helices are drawn to scale but not the connecting loops and turns. (E) Isolation of the two *psi* motifs in the DPBB structural fold. (F) A view similar to (C) of the solvent-accessible surface of *Pca*LOOL12 with the residues predicted to form a polysaccharide binding groove colored cyan or blue (A). The three labeled blue residues are associated with activity in expansins. (G) A view identical to (F) highlighting the electrostatic potentials at the solvent-accessible surface of *Pca*LOOL12 (–5 to +5 kT/e) with the negative and positive regions colored red and blue, respectively.

The elements of secondary structure observed in the solution structure of *Pca*LOOL12 are summarized in its primary amino acid sequence in Figure 1A and labeled on the tertiary structure of the protein in the ensemble in Figure 1B and the single structure closest to the average in Figure 1C (rotated 180 ° in the y-axis relative to the ensemble). Figure 1D is a topology diagram to assist visualization of the organization of the elements of secondary structure, six $\beta$-strands and three $\alpha$-helices, in the tertiary structure. The six $\beta$-strands hydrogen bond in a continuous fashion to form a barrel-like structure composed of two similar, three-strand units distinguished in blue and purple in the topology diagram. As illustrated in Figure 1E, three $\beta$-strands form a *psi*-motif composed of two relatively long antiparallel $\beta$-strands with the C-terminal of the C-terminal strand bent significantly relative to the N-terminal of the N-terminal strand, with a short strand running parallel to the C-terminal of the C-terminal strand (65). As illustrated best in Figure 1D, the two *psi*-motifs (*psi-1*: β6-β1-β5 (↓↑↑); *psi-2*: β3-β4-β2 (↓↑↑)) are positioned relative to each other to allow the short strands, β5 and β2, to form an anti-parallel pair to interlock the *psi*-motifs with an overall pseudo-twofold axis between the motifs. Such a fold is called a double-psi β-barrel (DPBB) and has been observed in a number of unrelated proteins including aspartate-α-decarboxylase, dimethyl sulfoxide reductase, barwin, endoglucanase V, and an endo-chitosanase (65, 66). Around the core DPBB fold are three α-helices, with the largest one, α2, nestled against the core and the two smaller ones part of loops extending from the core. Note that the largest loop, between β1 and β2, is tethered to the DPBB core fold at the N-terminal of β-3 via a disulfide bond, C26-C50 (Figure 1C, red). A second disulfide bond, C73-C76, is present in the loop between β4 and β5 with C73 forming part of the predicted polysaccharide binding groove (Figure 1F). Figure 1F also shows that most of the residues predicted to form a surface accessible polysaccharide binding groove (cyan and blue underlined residues in Figure1A) are surface exposed in the three-dimensional structure of *Pca*LOOL12 (residues A70 and M83 are buried in the middle of the protein and G50 is on the opposite face). Figure 1G shows that the surface of this face of the protein is negatively charged. A search for structures similar to *Pca*LOOL12 using the Dali server (67) identified seven structures with a Z-score greater then 8.5, six of these with a Z-score greater than 10 (Figure S3). In addition to expansin-related proteins, the structures include a protein annotated as a papain inhibitor from *Streptomyces mobaraensis* (5NTB) (68), hypothetical protein Pa4485 from *Pseudomonas aeruginosa* (4AVR) (72), kiwellin (4X9U) (69), the most abundant protein found in gold kiwifruit (*Actinidia chinensis*) (70), and an auxiliary metabolic gene (AMG) product (V-

Csn; 7TVL) displaying endo-chitinase activity from a soil phage identified in a metagenomic survey (71).

Previously, the 12 loosenins in the *P. carnosa* genome were divided into two subgroups, A and B, based on the position of an insertion relative to the predicted polysaccharide binding groove (15). *Pca*LOOL12 belongs in subgroup B due to a 14-residue insertion outlined in the red box in Figure 1A. On the other hand, *P. carnosa* loosenin classified into subgroup A have an 8-9 residue insertion at the position marked with an asterisk (Figure 1A). It was hypothesized that these insertions might confer different polysaccharide binding specificity for these two subgroups of loosenins (15). In the subgroup B *Pca*LOOL12 structure presented here, the C-terminal five residues of the insertion are part of a β-strand in the DPBB fold with the rest of the residues unstructured on the opposite face of the predicted polysaccharide binding groove (Figure 1F). Although it is difficult to imagine this region directly influencing the polysaccharide binding groove, the absence of the subgroup B insertion could impact the relative performance of loosenins in yet to be discovered ways. The location of the insertion in subgroup A loosenins is between β-strands β2 and β3, C-terminal to α1 in Figure 1C. Such an insertion may be able to fold into the predicted polysaccharide binding groove or extend the groove's breadth to effect polysaccharide binding specificity. An experimental structure of at least one *P. carnosa* subgroup A loosenin is necessary to distinguish these possibilities.

Temperature dependent NMR and circular dichroism (CD) experiments on *Pca*LOOL12 showed the protein to be stable to high temperatures. Figure S4 shows a series of $^{1}H$-$^{15}N$ HSQC spectra collected on the same 0.2 mM sample of $^{15}N$-labeled *Pca*LOOL12 at 20 °C, 60 °C, and back at

20 °C. While the spectrum at 60 °C contains only a few poorly dispersed amide cross peaks suggestive of an unfolded protein, after cooling the sample back to 20 °C the $^{1}H$-$^{15}N$ HSQC spectrum is very similar to the spectrum collected prior to heating (there is evidence of some new resonances appearing above the noise suggesting the refolding is not 100% efficient). Similar CD experiments performed on a 0.02 mM sample of *Pca*LOOL12 from 10 to 80 °C also suggested *Pca*LOOL12 unfolded at high temperature and refolded after heating (data not shown). Note further that there was no visible evidence of protein precipitation after heating either in a CD cell or NMR tube. Thermal stability (Tm = 85 °C) was also reported for the papain inhibitor from *S. mobaraensis* (5NTB) that is structurally similar to *Pca*LOOL12 (Figure S2) (68,72). Both proteins contain two disulfide bonds in the same relative positions and disulfide bonds are known to increase the stability of proteins and peptides (73). While additional experiments will be necessary to confirm if these disulfide bonds are responsible for the observed thermal stability, they are likely not required to stabilize the tertiary structure of *Pca*LOOL12 as its structure is very similar to the structure of *P. aeruginosa* Pa4485 (4AVR, Figure S3) despite the absence of any cysteine residues in the latter protein.

The structure of *Pca*LOOL12 produced in *P. pastoris* was then evaluated by size-exclusion chromatography coupled with small-angle X-ray scattering (SEC-SAXS). The SEC-SAXS profile showed a single peak between SAXS frames 209 and 256, and the radius of gyration ($R_g$) values calculated from the individual SAXS profiles showed a decrease from approximately 18 Å to 15 Å across the elution peak (Figure S5). Evolving Factorial Analysis (EFA) was used to deconvolute the different species in the SEC-SAXS profile and identified *Pca*LOOL12 oligomers with calculated $R_g$ values of 15.7 ± 0.2 Å and 23.2 ± 0.6 Å (Table 1). The smaller component was consistent with the NMR structure of the *Pca*LOOL12 produced in *E. coli*, a monomer with a molecular weight (MW) of 12.6 kDa. The larger MW species is consistent with a *Pca*LOOL12 dimer (MW = 24.5 kDa). The slightly larger MW of the *Pca*LOOL12 monomer obtained by SEC-SAXS (12.6 kDa) compared to NMR (10.8 kDa) is due to the presence of the C-terminal $His_6$-tag in the former. Despite this, the NMR conformers provided a reasonably good fit to the SAXS data ($\chi^2$ of 3.60 and 7.28 for the NMR conformer with the best and worst fit, respectively). Notably, the AlphaFold2 *Pca*LOOL12 structure is very similar to that of NMR structure that best fit the SAXS data ($\chi^2$ of 3.60). The backbone root-mean-square deviation (RMSD) between the AlphaFold2 and NMR structures is 0.71 Å for residues 1 to 103, excluding the C-terminal $His_6$-tag present in the AlphaFold2 model (Figure S6). Using the AlphaFold2 *Pca*LOOL12 model that included the $His_6$-tag yielded a SAXS fit of $\chi 2 = 4.8$, indicating that the addition of the $His_6$-tag alone does not account for the protein's structural flexibility observed in SAXS (Figure 2). Normal Mode Analysis (NMA) was then used to calculate flexible motions within the protein, which provided a conformation of *Pca*LOOL12 with NMA $\chi 2$ value of 1.5 (Figure 2). The RMSD for the backbone between the NMA and NMR models was 1.40 Å for residues 1 to 103. Most of the significant differences in the amino acid backbone RMSD were observed in the N-terminal region (residues 1 – 12) (Figure S7), which was also identified as a highly flexible region in our NMR structure. This highly flexible N-terminal, combined with the presence of the $His_6$-tag, provided good agreement between the NMA model and the SAXS data.

Although NMR structures were not determined for *Pca*LOOL7 and *Pca*LOOL9, the agreement between the SEC-SAXS derived model of *Pca*LOOL12 and its NMR structure supports the structural assessment of *Pca*LOOL7 and *Pca*LOOL9 using SEC-SAXS alone. The elution profile of *Pca*LOOL7 showed a single peak between SAXS frames from 180 to 220, and the $R_g$ values calculated from the individual SAXS profiles showed a decrease from 17.8 Å to 13.7 Å across the elution profile (Figure S5). Using EFA to deconvolute the SEC-SAXS profile, two major components were obtained with $R_g$ values of 17.1 ± 0.1 Å and 13.4 ± 0.3 Å (Table 1). The larger species is consistent with a *Pca*LOOL7 monomer with a MW of 14.9 kDa; the smaller particle had a MW of 11.1 kDa and was likely due to proteolytic degradation of the holoprotein. The predicted AlphaFold2 model fit the SAXS data of *Pca*LOOL7 reasonably well ($\chi 2$ of 6.0) (Figure S8) and improved using NMA (NMA $\chi 2$ value of 1.6) (Figure 2).

A similar approach was used to analyze *Pca*LOOL9. In this case, the SEC-SAXS profile also showed a single peak between SAXS frames 160 and 220 (Figure S5). The $R_g$ values calculated from the individual frames decreased from approximately 67 Å to 20 Å across the elution peak. EFA also showed the presence of *Pca*LOOL9 oligomers with MW values ranging between 320 kDa and 18.8 kDa, a considerably wider range than observed for *Pca*LOOL12 and *Pca*LOOL7. The $R_g$ values of the SAXS frames plateau between frame 200 – 220 and the calculated MW and $R_g$ were 11.9 kDa and 15.7 ± 0.1 Å (Table 1), respectively, consistent with a *Pca*LOOL9 monomer. Unlike *Pca*LOOL12 and *Pca*LOOL9, the predicted AlphaFold2 model did not provide a good fit to the SAXS data of *Pca*LOOL9 ($\chi 2$ of 31.7) (Figure S8); however, NMA provided conformations that fit the data for *Pca*LOOL9 with a $\chi 2$ value of 2.5 (Figure 2). Notably, the structures generated from NMA for all three *Pca*LOOL proteins were similar to the AlphaFold2 models except for the N-terminal region (1 – 26) and the unstructured C-terminus $His_6$-tag, which were predicted with relatively low confidence (predicted local distance difference test (pLDDT) between 50 – 70%).

**Table 1:** Structural parameters and data fitting of *Pca*LOOL proteins from SEC-SAXS

| | ***Pca*LOOL7** | ***Pca*LOOL9** | ***Pca*LOOL12** |
|---|---|---|---|
| **Guinier analysis** | | | |
| $I$ (0) (a.u) | 109.7± 0.3 | 93.5± 0.1 | 82.7± 0.4 |
| $R_g$ (Å) | 17.1 ± 0.1 | 15.7 ± 0.1 | 15.7 ± 0.2 Å |
| $Q$x$R_g$ range | 0.23 - 1.29 | 0.30 – 1.30 | 0.20 - 1.28 |
| **$P$(r) analysis** | | | |
| $I$ (0) (a.u) | 109.8 ± 0.3* | 94.6 ± 0.2 | 83.8 ± 0.5 |
| $R_g$ (Å) | 17.9 ± 0.2 | 16.4 ± 0.1 | 16.5 ± 0.3 |
| $D_{max}$ (Å) | 72 | 91 | 60 |
| $Q$ range (Å$^{-1}$) | 0.14–0.5 | 0.013–0.4 | 0.012–0.3 |
| $\chi^2$ | <1.0 | 1.0 | 1.2 |
| **Molecular weight ($M_W$) analysis** | | | |
| $M_W$ from Porod volume (kDa) | 14.9 | 11.9 | 12.6 |

* *I (0)* from SEC-SAXS is not on absolute scale; Theoretical molecular weights (calculated from their primary amino acid sequences) of *Pca*LOOL7, *Pca*LOOL9, and *Pca*LOOL12 are 13.44 kDa, 11.98 kDa, and 11.4 kDa, respectively.

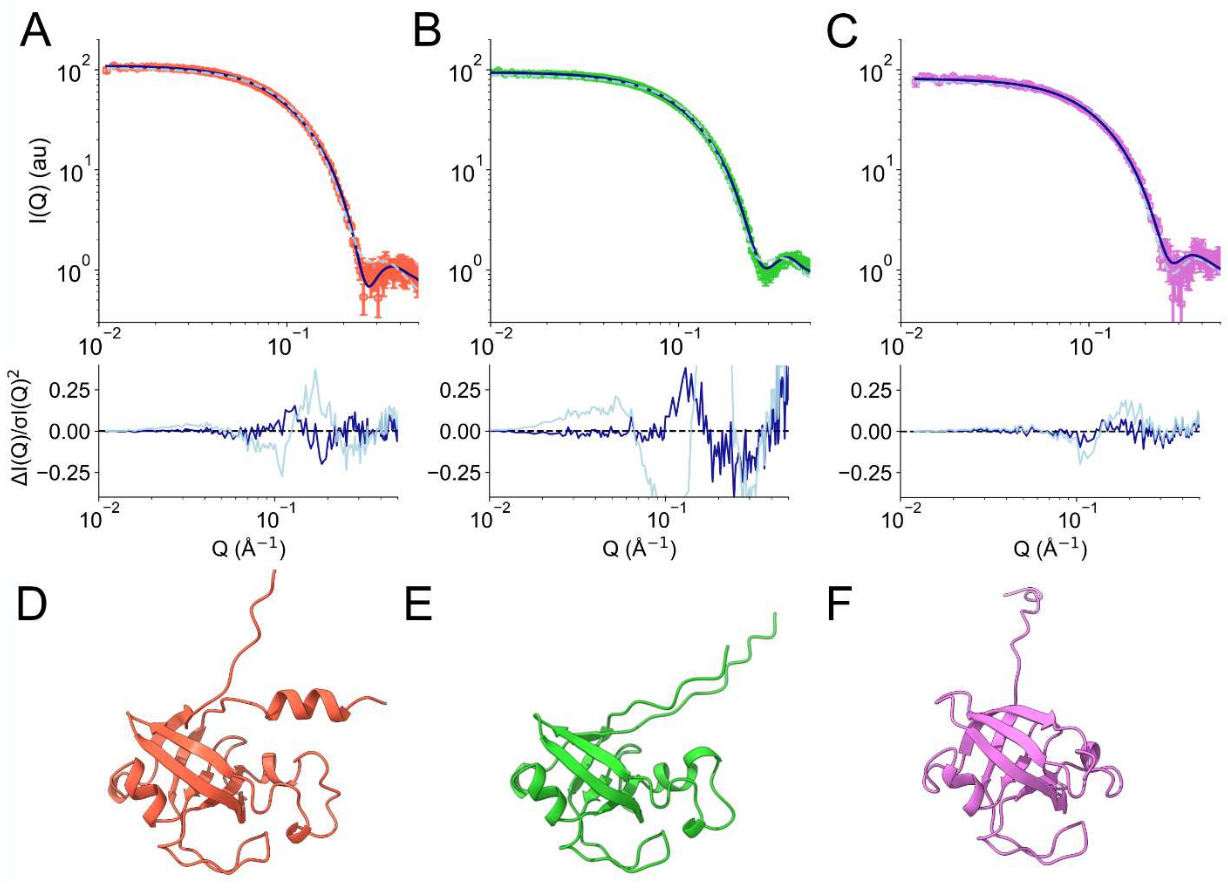

**Figure 2**. Structural analysis of *Pca*LOOLs. Panels A to C) Experimental SAXS profiles of *Pca*LOOL7 (orange), *Pca*LOOL9 (green), and *Pca*LOOL12 (pink) are shown as open hexagons. Theoretical SAXS profiles from AlphaFold2 and Normal Mode Analysis are shown as dashed light and solid dark blue lines, respectively. Error-normalized residual plots are show below the SAXS profiles. Panels D to F) Normal Mode Analysis models of *Pca*LOOL7 (orange), *Pca*LOOL9 (green), and *Pca*LOOL12 (pink).

**Characterization of native wood samples and holocelluloses prior to *Pca*LOOL treatment.**

Compositional analyses of native and delignified Eucalyptus and Spruce samples confirmed the efficacy of the sodium hypochlorite delignification treatment (Figure 3, Table S3). Specifically, after 6 h, the sodium hypochlorite treatment reduced the lignin content of Eucalyptus samples by 87%, from 36 wt% to 5 wt% of the total sample. Similarly, the lignin content of Spruce samples was reduced by 75%, from 28 wt% to 8 wt% of the total sample. In parallel, the monosaccharide composition revealed that the delignification process did not significantly alter the cellulose and hemicellulose populations in the Eucalyptus and Spruce wood samples. The Eucalyptus holocellulose fibers showed a cellulose content of approximately 57 wt%, retaining a hemicellulose content of 18 wt%, in which (galacto)glucuronoxylan was, as expected, the most dominant component with a negligible content of glucomannan (< 2 wt%). In parallel, the Spruce holocellulose fibers contain approximately 59 wt% of cellulose and retained a total hemicellulose content of approximately 29 wt%, where galactoglucomannan was the most abundant component (18 wt%) with a significant content of arabinoglucuronoxylan (11 wt%).

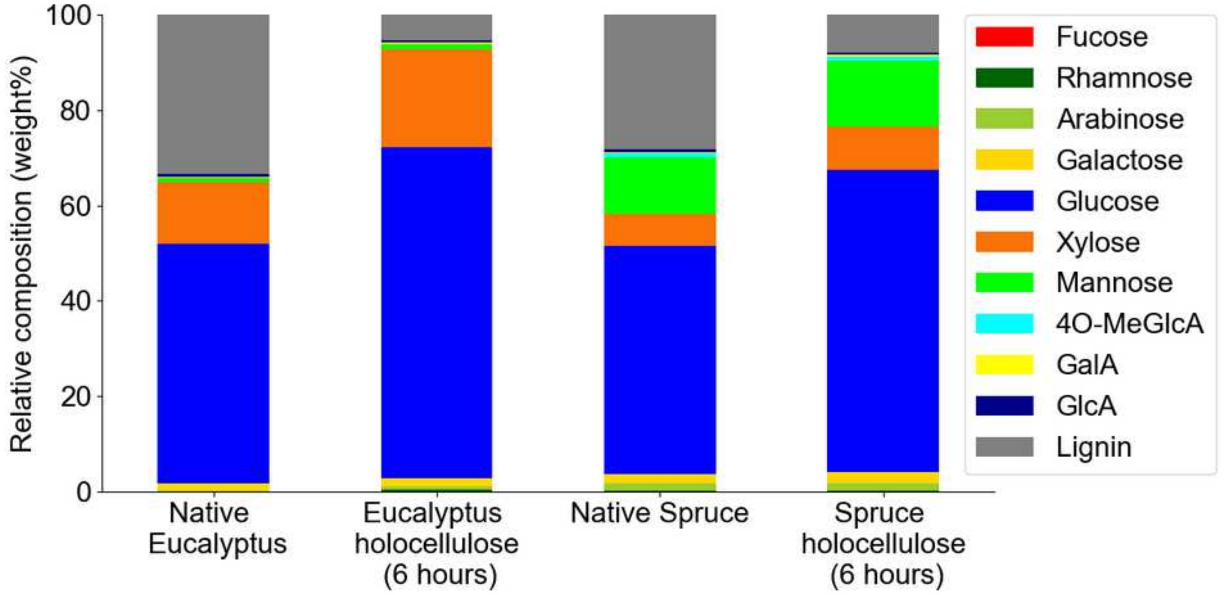

**Figure 3.** Relative monosaccharide and lignin composition of untreated wood (Eucalyptus and Spruce) and holocellulose samples. Values represent the relative weight of the corresponding monosaccharide from acid hydrolysis and the measured lignin after the acetyl bromide dissolution in the total weight of the biomass sample.

The impact of partial delignification and varying hemicellulose contents on holocellulose structure was investigated using SANS. Especially, equatorial scattering is of importance where overall intensity in the equatorial region (low Q values) of the SANS data can point to cellulose microfibrillar morphology and order aligned along the stem growth direction as well as the morphology of nanopores within the wood cell wall in some cases (74, 75). By contrast, the meridional (or vertical) scattering region is dominated by the amorphous components in the cell wall such as residual lignin, hemicellulose, and cellulose microfibrils aligned parallel to the direction of the neutron beam (59).

The 2D SANS scattering patterns of native Eucalyptus and Spruce (Eu_native, Sp_native) and holocellulose (Eu_HC, Sp_HC) are shown in Figure 4A-D. The differences in the images can be attributed to the changes in the organization of the aligned cellulose microfibrils in the cell walls. Both native Eucalyptus and Spruce generated a similar 2D scattering pattern; a sharp streak-like pattern in the equatorial direction of the main detector and a lobe-like feature extended into the wing detector image. This indicates that the cellulose microfibrils were well-aligned along the growth direction of the plants (59,76). In contrast, the 2D scattering patterns of the holocellulose samples were less defined compared to the native samples; moreover, the lobe feature in the wing detector was missing indicating that the cellulose microfibril ordering decreased after lignin removal.

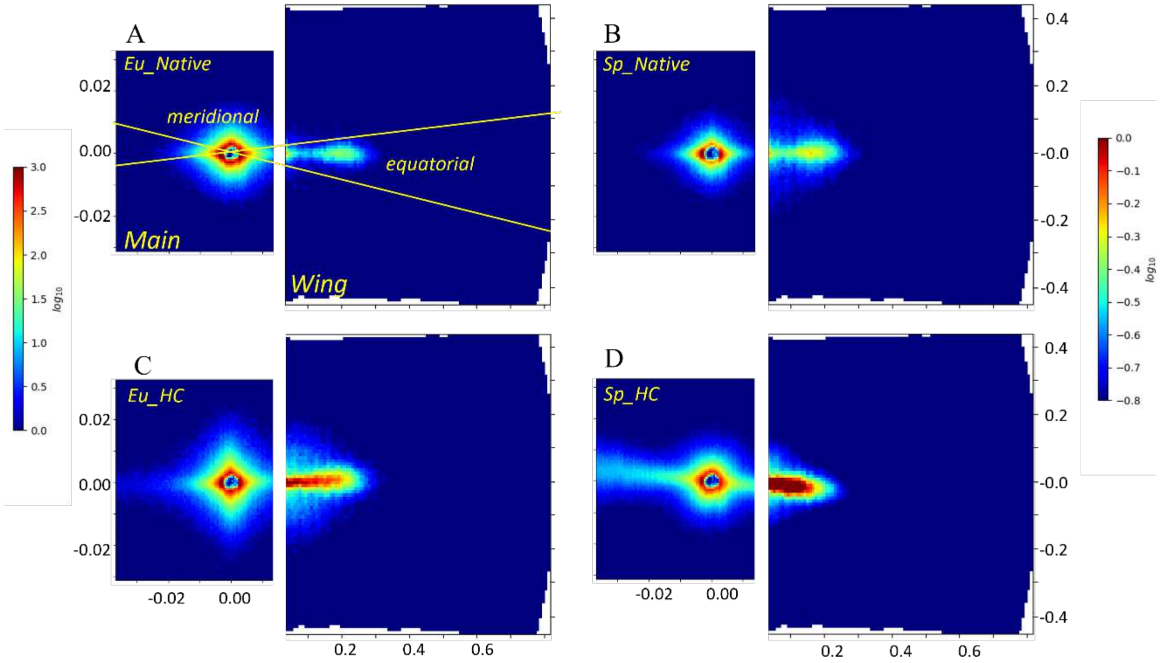

**Figure 4**. Two-dimensional SANS detector images of native Eucalyptus (A) and Spruce (B), and holocellulose from Eucalyptus (C) and Spruce (D) in 20 mM sodium acetate buffer, pH 5.5 in 100% $D_2O$. Each panel shows the main (left) and wing (right) detector images from the SANS instrument. Lines in the panel A represent the equatorial and meridional sectors that were used for data analysis and interpretation.

The 2D scattering patterns were reduced to 1D scattering profiles using the wedge reduction approach as described in the Materials and Methods to obtain equatorial and meridional curves of the native samples (grey profiles in Figure 5 and Figure 6, respectively). The equatorial profiles showed a well-defined peak in the high Q region (Q ~ 0.05 – 0.4 $Å^{-1}$) and specifically at Q ~ 0.2 $Å^{-1}$ that is attributed to the radial spacing between cellulose microfibrils aligned along the growth direction of the plants. In the low Q region (Q ~ 0.003 – 0.05 $Å^{-1}$), both native species exhibit a steep monotonic increase in intensity for decreasing Q values, which is attributed to the existence of large structures (*d* of 0.1 – 0.2 μm) such as cellulose macrofibrils or cell wall lumen (Figure 5).

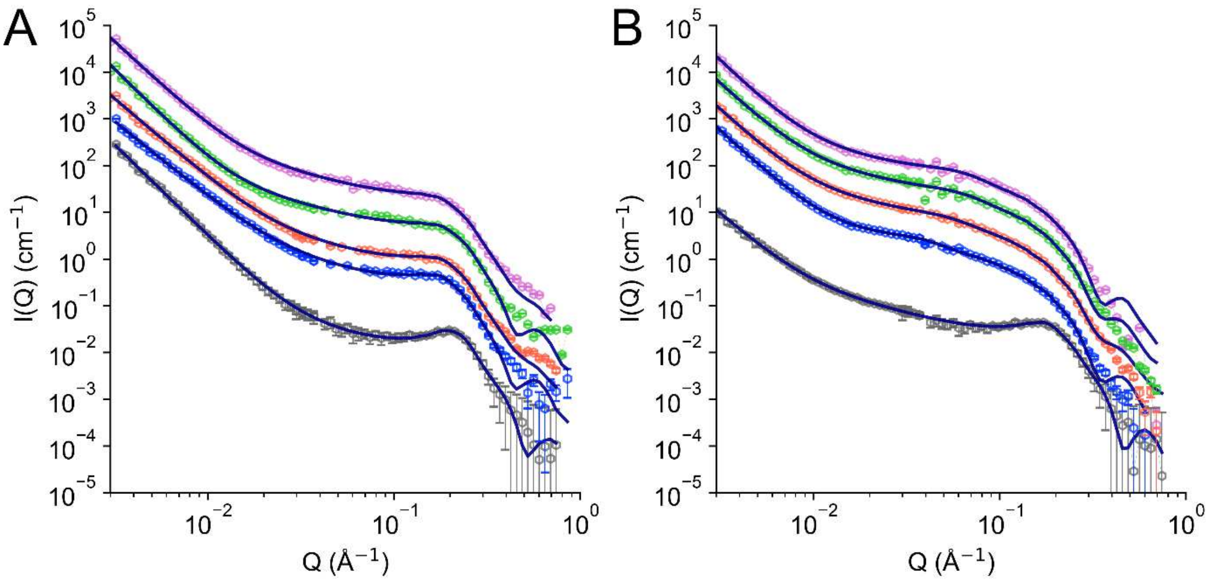

**Figure 5.** One-dimensional equatorial SANS profiles of Eucalyptus (A) and Spruce (B). The color labeling schemes for the left and right panels are identical. The holocellulose samples were incubated for 24 h with the *Pca*LOOL proteins before SANS measurements. Native wood sample (grey); holocellulose control (blue); *Pca*LOOL7 (orange); *Pca*LOOL9 (green), *Pca*LOO12 (pink). The *Pca*LOOL7, *Pca*LOOL9, and *Pca*LOOL12 curves were multiplied by multiples of 4, and native wood sample was multiplied by a factor of 0.16 for data visualization purposes.

Quantitative analysis of the equatorial SANS profiles was performed using a model that comprises of a cylindrical form factor coupled with a structure factor to model the high Q signature (Q ~ 0.05 – 0.4 $Å^{-1}$), and a power-law for the low Q region (Q ~ 0.003 – 0.05 $Å^{-1}$). The fitting parameters are listed in Table 2. The cross-sectional radius of the cylindrical particles, representing cellulose microfibrils of native Eucalyptus and Spruce samples are 7.9 ± 0.7 Å and 8.3 ± 0.7 Å, respectively, which agrees well with previously reported values (29). The peak feature in the high-Q region (0.05 – 0.4 $Å^{-1}$) provides a measure of the center-to-center distance of neighboring cellulose microfibrils ($d_{spacing}$). The $d_{spacing}$ value of 30 ± 3 Å for Spruce is consistent with those previously reported for Sitka spruce (3-4 nm depending on moisture content) (77). The $d_{spacing}$ value of 26 ± 1 Å for Eucalyptus, however, was somewhat lower than reported values for other angiosperms including birch (3.4 nm) and aspen (4 nm) (78, 79). The generally higher microfibril spacing in angiosperms over gymnosperms has been attributed to the higher xylan content in angiosperms and regular binding of the xylan to hydrophilic faces of microfibrils (80–82), which could putatively accommodate a monolayer of xylan chains bound to the cellulose surfaces in a similar 2-fold screw conformation. Although we cannot rule out impacts of sample age and storage conditions of the Spruce and Eucalyptus wood chips used herein, the comparatively low microfibril spacing (i.e., $d_{spacing}$ value) measured for Eucalyptus could reflect the structure of Eucalyptus xylan, in which the presence of galactose substitutions covalently bound to 4-*O*-methyl glucuronic acid decorations (82) might hinder xylan interactions with cellulose surfaces.

The equatorial SANS profiles do not provide a scattering signature for macrofibrils because the same water and amorphous polymer (lignin and hemicellulose) contrast exists between individual microfibrils and the bundles that comprise the macrofibrils. Moreover, the loosely defined boundary for macrofibrils potentially contribute to a larger polydispersity in the cross-sectional radius of the macrofibrils making it difficult to observe. Nevertheless, the packing parameter (k) estimates the degree of packing of cellulose microfibrils in the wood samples (64). The most efficient packing arrangement, such as a hexagonally packed lattice or face-centered cubic lattice, has a k value of 5.92 that decreases to zero in a random distribution of particles

(62). The packing parameter is 4.6 ± 0.2 and 3.6 ± 0.2 for Eucalyptus and Spruce, respectively indicating that the cellulose microfibrils have a regular arrangement in both native samples (Table 2).

The equatorial scattering profiles of the Eucalyptus and Spruce holocellulose were significantly different from the native sample (Figure 5). The most visible change was the loss of the strong peak feature in the high-Q region which represents the loss of regular order in the arrangement of the cellulose microfibrils. This is consistent with the observed loss of a strong lobe-like feature in the 2D patterns upon delignification. The cross-sectional radius of the cellulose microfibril did not show appreciable change on delignification for either Eucalyptus or Spruce, consistent with the potential lignin localization in the outer parts of macrofibrils (83). In contrast, the values for the $d_{spacing}$ in the Eucalyptus samples increased by 15 % from 26 Å to 30 Å; the increase was more significant in Spruce where the $d_{spacing}$ values increased by 190 %, from 30 Å to 87 Å (Table 2). The increase in center-to-center distance between neighboring cellulose microfibrils was coupled with a decrease in the degree of order (i.e., packing parameter, $f_{pack}$), where $f_{pack}$ reduced from 4.6 to 2.8 in Eucalyptus and from 3.6 to 0.7 in Spruce (Table 2). The more significant impacts of delignification on the regular arrangement of the cellulose microfibrils in Spruce compared to Eucalyptus could be caused by a reorganisation of the corresponding hemicelluloses (i.e., mainly the accessible acetylated galactoglucomannan populations in Spruce) (84). This aligns well with the observation that softwood macrofibrils may be more sensitive to drying than hardwood macrofibrils (85), indicating that the former could be more accessible and thus more affected by delignification treatments, and potentially, the activity of loosenins.

**Table 2.** Fitting parameters extracted from the equatorial SANS data analysis.

| Sample | Cylinder | | | | Power-Law Exponent |
|---|---|---|---|---|---|
| | Volume Fraction | $R_{cs}$ (Å) | $d_{spacing}$ (Å) | $f_{pack}$ | P |
| Q range (Å$^{-1}$) | 0.05-0.4 | | | | 0.003- 0.04 |
| Eu_native | 0.051 ± 0.007 | 7.9 ± 0.7 | 26 ± 1 | 4.6 ± 0.2 | 3.8 ± 0.2 |
| Eu_HC | 0.094 ± 0.001 | 8.7 ± 0.9 | 30 ± 1 | 2.8 ± 0.3 | 3.6 ± 0.2 |
| Eu_*Pca*LOOL12 | 0.079 ± 0.002 | 8.6 ± 1.5 | 29 ± 2 | 1.8 ± 0.2 | 3.7 ± 0.1 |
| Eu_*Pca*LOOL7 | 0.060 ± 0.001 | 8.7 ± 1.6 | 29 ± 1 | 2.6 ± 0.3 | 3.6 ± 0.2 |
| Eu_*Pca*LOOL9 | 0.078 ± 0.001 | 8.4 ± 0.7 | 29 ± 1 | 2.2 ± 0.2 | 3.9 ± 0.1 |
| | | | | | |
| Sp_native | 0.055 ± 0.001 | 8.3 ± 0.7 | 30 ± 3 | 3.6 ± 0.2 | 3.5 ± 0.1 |
| Sp_HC | 0.039 ± 0.002 | 10.9 ± 1.4 | 87 ± 6 | 0.7 ± 0.1 | 3.8 ± 0.1 |
| Sp_*Pca*LOOL12 | 0.051 ± 0.001 | 10.9 ± 1.4 | 138 ± 10 | 0.7 ± 0.1 | 3.7 ± 0.1 |
| Sp_*Pca*LOOL7 | 0.055 ± 0.001 | 10.9 ± 2.1 | 103 ± 8 | 0.5 ± 0.2 | 3.7 ± 0.2 |
| Sp_*Pca*LOOL9 | 0.054 ± 0.001 | 11.1 ± 1.7 | 88 ± 4 | 0.6 ± 0.1 | 3.7 ± 0.1 |

Eu: Eucalyptus; Sp: Spruce; HC: holocellulose

The SANS data in the meridional sector of the 2D pattern arise from the isotropic scattering features in the plant cell wall such as the cellulose microfibrils aligned parallel or close to parallel to the neutron beam direction, and the amorphous matrix of lignin and hemicellulose co-polymers. The high Q region (Q ~ 0.075 – 0.4 Å$^{-1}$) was modelled to a spherical form factor and a power-law function in the intermediate Q region (Q ~ 0.01 – 0.075 Å$^{-1}$) (Figure 6). The spherical form factor represents the isotropic scattering feature of the cellulose microfibril aligned parallel to the neutron beam. The cross-sectional radius of the cellulose microfibril assemblies were similar for the native species, 9.3 ± 2.2 Å for Eucalyptus and 11.9 ± 1.0 Å for Spruce; however, the power-law exponent that represent structural organization in the length scale of 8-60 nm were different (Table 3). An exponent of approximately -2 (-2.1 ± 0.1) for the Eucalyptus samples indicates a randomly flexible conformation of the matrix co-polymers implying more overlapping and criss-crossing conformations, while an exponent of -1.4 (-1 to -2) for Spruce samples indicates a matrix co-polymer conformation having limited flexibility and less overlapping (86). SANS data in the low Q region (Q ~ 0.003 – 0.01 Å$^{-1}$) was similar in both samples, exhibiting a power-law exponent of approximately -4 which suggests the presence of micron-sized assemblies.

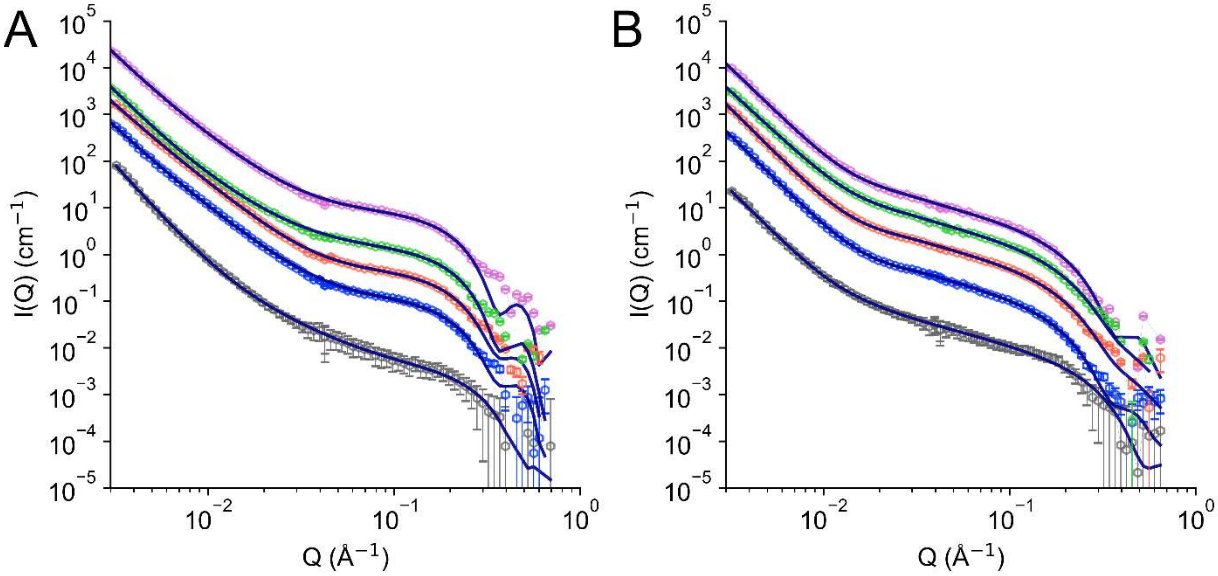

**Figure 6.** One-dimensional meridional SANS profiles of Eucalyptus (A) and Spruce (B). The color labeling schemes for the left and right panels are identical. The holocellulose samples were incubated for 24 h with the *Pca*LOOL proteins before SANS measurements. Native wood sample (grey); holocellulose control (blue); *Pca*LOOL7 (orange); *Pca*LOOL9 (green), and *Pca*LOO12 (pink). The *Pca*LOOL7 , *Pca*LOOL9, and *Pca*LOOL12 curves were multiplied by multiples of 4, and native wood sample was multiplied by a factor of 0.16 for data visualization purposes.

Delignification led to an increase in the cellulose microfibril cross-sectional radius from 9.3 ± 2.2 Å to 12.4 ± 0.2 Å for Eucalyptus and from 11.9 ± 1.0 Å to 19.7 ± 1.0 Å for Spruce (Table 3, Figure 6). The higher extent of increase in the cross-sectional radius for Spruce than for Eucalyptus indicates a higher degree of misaligned cellulose microfibrils from the parallel alignment to the neutron beam. This is consistent with the higher degree of loss of order for Spruce as also measured from the equatorial sector, again suggesting greater impact of delignification on the mobility of the remaining hemicellulose. At the same time, the matrix co-polymer conformation remained unchanged between native and delignified Eucalyptus and Spruce samples. A lower exponent of -1.4 for Spruce, compared to -2.0 for Eucalyptus, will have no overlapping and interpenetrating conformations in the amorphous biopolymers, which results in reduced constraints in maintaining order in the cellulose microfibril arrangement after delignification. On the other hand, random flexible conformations which represents overlapping and interpenetrating conformation as observed in Eucalyptus will still contribute to maintaining order in the cellulose microfibril arrangement even after delignification. These holocellulose samples with varying lignin content after the delignification process, and with retained hemicellulose content and distinct hemicellulose populations in terms of glucomannan and xylan depending on the source, represent valuable substrates to investigate the distinct effect of the different loosenins in disrupting non-covalent interactions between the cellulose microfibrils and the different matrix polysaccharides.

**Table 3.** Fitting parameters extracted from the meridional SANS data analysis.

| Sample | Sphere | | Power-Law Exponent | |
|---|---|---|---|---|
| | Volume Fraction | $R_{sph}$ *(Å)* | P2 | P3 |
| Q range (Å$^{-1}$) | 0.075 – 0.4 | | 0.01 – 0.075 | 0.003 – 0.01 |
| Eu_native | 0.004 ± 0.002 | 9.3 ± 2.2 | 2.1 ± 0.1 | 4.2 ± 0.2 |
| Eu_HC | 0.009 ± 0.003 | 12.4 ± 0.2 | 2.0 ± 0.2 | 4.0 ± 0.2 |
| Eu_*Pca*LOOL12 | 0.011 ± 0.002 | 12.3 ± 0.1 | 2.2 ± 0.1 | 4.1 ± 0.1 |
| Eu_*Pca*LOOL7 | 0.009 ± 0.003 | 12.4 ± 0.1 | 2.1 ± 0.1 | 4.1 ± 0.1 |
| Eu_*Pca*LOOL9 | 0.007 ± 0.003 | 12.3 ± 0.1 | 2.0 ± 0.2 | 4.0 ± 0.1 |
| | | | | |
| Sp_native | 0.008 ± 0.002 | 11.9 ± 1.0 | 1.4 ± 0.2 | 3.9 ± 0.2 |
| Sp_HC | 0.008 ± 0.002 | 19.7 ± 0.1 | 1.3 ± 0.3 | 4.0 ± 0.2 |
| Sp_*Pca*LOOL12 | 0.003 ± 0.001 | 18.1 ± 0.1 | 1.4 ± 0.1 | 4.0 ± 0.1 |
| Sp_*Pca*LOOL7 | 0.005 ± 0.003 | 18.6 ± 0.1 | 1.4 ± 0.1 | 4.1 ± 0.1 |
| Sp_*Pca*LOOL9 | 0.003 ± 0.001 | 19.4 ± 0.1 | 1.4 ± 0.1 | 4.0 ± 0.1 |

Eu: Eucalyptus; Sp: Spruce; HC: holocellulose

**Effect of *Pca*LOOLs on native wood and holocellulose samples.** The 1D equatorial SANS profiles of Eucalyptus and Spruce holocellulose samples after 24 h reaction with different *Pca*LOOL proteins are shown in Figure 5. Quantitative structural information extracted from the fitted data (Table 2) revealed no change to the microfibril cross-sectional radius after treatment with *Pca*LOOLs, indicating the proteins do not induce detectable fiber swelling. Instead, *Pca*LOOLs impacted the inter-microfibril distance ($d_{spacing}$) and the packing parameter ($f_{pack}$) of holocelluloses in a substrate-dependent manner (Figure 7).

Specifically, *Pca*LOOL12 increased the $d_{spacing}$ in Spruce holocellulose by 59%, from 87 ± 6 Å to 138 ± 10 Å, and *Pca*LOOL7 increased the $d_{spacing}$ by 18%, from 87 ± 6 Å to 103 ± 8 Å (Figure 7A, Table 2). Although *Pca*LOOL treatment did not impact $d_{spacing}$ in Eucalyptus holocellulose, *Pca*LOOL12 reduced the $f_{pack}$ for Eucalyptus holocellulose by 36% from 2.8 ± 0.3 to 1.8 ± 0.2, and *Pca*LOOL9 reduced the $f_{pack}$ by 21% from 2.8 ± 0.3 to 2.2 ± 0.2 (Figure 7B, Table 2).

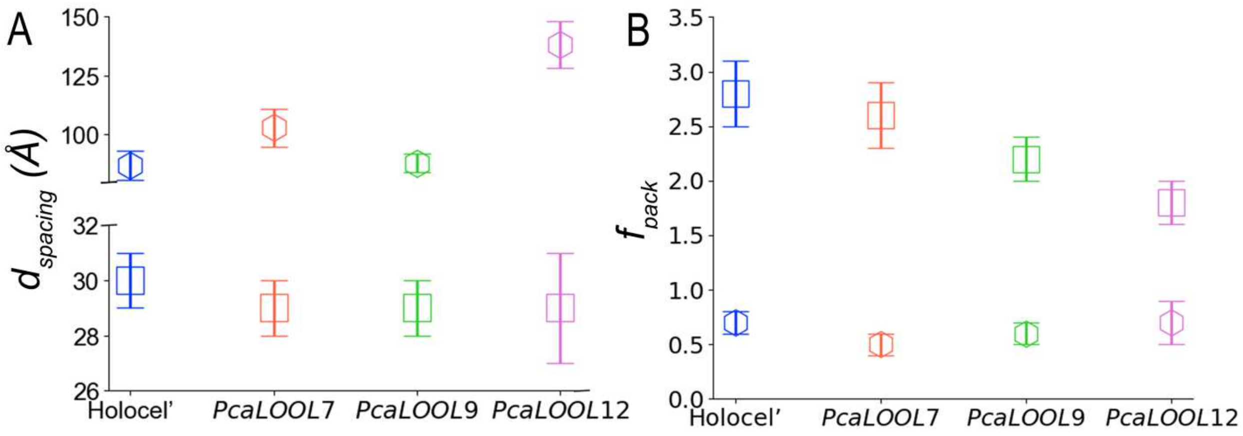

**Figure 7.** Inter-microfibril distance (A) and packing parameter (B) obtained from fitting the Eucalyptus (open squares) and the Spruce (open hexagons) data.

The meridional scattering profiles for the series of samples treated with *Pca*LOOLs showed no change, implying that *Pca*LOOLs mainly affected the regular arrangement of the cellulose microfibrils and not the cross-sectional radius of the cellulose microfibrils, micron-sized large structures, or the matrix co-polymer organization (Table 3, Figure 6).

The impact of *Pca*LOOL12, and to a lesser extent *Pca*LOOL7, on the $d_{spacing}$ of Spruce holocellulose but not Eucalyptus holocellulose correlates to the larger impact that delignification alone had on the $d_{spacing}$ of Spruce microfibrils (Table 2). Since the lignin is expected to largely localize to the outer parts of macrofibrils, the reorganization of hemicelluloses (galactoglucomannan) upon the delignification of Spruce could have induced the observed increase in $d_{spacing}$ of microfibrils, which could increase *Pca*LOOL12 access to targeted regions within the microfibrils. Delignification had a comparatively low impact on the inter-fibril spacing ($d_{spacing}$) of Eucalyptus microfibrils (Table 2). A compelling possibility, then, is that the decrease in $f_{pack}$ observed when treating Eucalyptus holocellulose with *Pca*LOOL12, and to a lesser extent *Pca*LOOL9, might precede protein-mediated impacts to $d_{spacing}$. Evaluating the impact of time and protein load would address this possibility. The greatest impact of *Pca*LOOL12 on holocellulose samples compared to other *Pca*LOOLs is intriguing and yet consistent with earlier studies that show comparatively high impacts of *Pca*LOOL12 on cellulose filter paper weakening and boosting of cellulolytic enzymes on lignocellulose (21,27). Notably, early transcriptomic studies of *P. carnosa* showed highest transcript abundance for *Pca*LOOL12 over all other loosenins encoded by the source organism, suggesting a comparatively important biological function for *Pca*LOOL12 (15). Moreover, *Pca*LOOL12 was assigned to a different phylogenetic subgroup than *Pca*LOOL7 and *Pca*LOOL9 (15), has a comparatively low predicted surface charge (27), and herein was observed to adopt the most compact protein conformation which could enhance its accessibility to targeted positions within cellulose substrates.

### CONCLUSIONS

Microbial expansin-related proteins, including loosenins, constitute a compelling protein family for opening up cellulose fiber networks to enable fiber dissolution or enzymatic treatments that lead to fiber surface modification or deconstruction. While this protein family is thought to act at tight junctions between neighboring cellulose microfibrils or between cellulose microfibrils and matrix polysaccharides, studies to date have been somewhat conflicted concerning impacts of the protein action on fiber swelling versus fibrillation. Herein, SANS was used to study the impact of loosenins from *P.carnosa* (*Pca*LOOL7, *Pca*LOOL9, and *Pca*LOOL12) on the organization of cellulose microfibrils in hardwood (Eucalyptus) and softwood (Spruce) following their partial delignification. *Pca*LOOL12 treatment led to greatest impacts on both Eucalyptus and Spruce samples, leading to a decrease in packing organization and increase in interfibril distance, respectively. Notably, compared to Eucalyptus, the delignification of the Spruce sample substantially reduced the packing organization of corresponding microfibrils prior to *Pca*LOOL treatment, likely due to differences in the corresponding type and content of hemicelluloses. Accordingly, the differences in impacts of *Pca*LOOL12 on these materials, suggests a progression of protein action from reducing microfibril packing order to increasing interfibril spacing. Critically, the microfibril radii were unchanged after *Pca*LOOL treatment, indicating the proteins induce fiber fibrillation over microfibril swelling; however, the possibility that microfibril swelling would be observed over time cannot be ruled out. The progression of *Pca*LOOL action on the holocellulose samples could be addressed through SANS studies that investigate impact of *Pca*LOOL12 dose and incubation time on the microfibril organization.

Structural characterization of the *Pca*LOOLs points to their functioning as monomeric proteins. In particular, the NMR analysis of *Pca*LOOL12 reveals the first solved structure of a loosenin protein, and thereby, significantly augments the small number of expansin-related protein structures currently available. The NMR structure shows the N-terminal insertion of *Pca*LOOL12 is located distal to the polysaccharide binding groove, and so it is difficult to see how the insertion could directly impact substrate interactions. Alternatively, it is conceivable that the sequence insertion in *Pca*LOOL7 and *Pca*LOOL9, which is lacking from *Pca*LOOL12 and shows potential to fold into or extend the predicted polysaccharide binding groove, could have limited their action on the holocelluloses tested herein. Structures of these subgroup A *Pca*LOOLs will help to examine this prediction, and combined with a broader analysis of loosenin model structures, will shed additional light on sequence and structural determinants of substrate preference within the expansin-related protein family.

**LIST OF ABBREVIATIONS**

LOOL: Loosenin-like proteins

EXLX1: microbial expansin-like proteins

SANS: small-angle neutron scattering

SEC SAXS: size exclusion chromatography small-angle x-ray scattering

WAXS: wide angle x-ray scattering

NMR: nuclear magnetic resonance

NOESY-HSQC: Nuclear Overhauser Effect Spectroscopy - Heteronuclear single quantum coherence

NMA: Normal Mode Analysis

HPAEC-PAD high-performance anion-exchange chromatography coupled with pulsed amper: metric detection

**ACKNOWLEDGMENTS**

We acknowledge all scientific discussions enabled through the BioUPGRADE research network (https://bioupgrade.eu).

**FUNDING**

This project has received funding from the European Union's Horizon 2020 research and innovation programme under grant agreement No 964764. The content presented in this document represents the views of the authors, and the Commission is not responsible for any use that may be made of the information it contains. This research was also performed on project award 60859 under the FICUS program ( https://dx.doi.org/10.46936/fics.proj.2023.60859/60008908) and used resources at the DOE Environmental Molecular Sciences Laboratory, which are DOE Office of Science User Facilities. The facility is sponsored by the Biological and Environmental Research program and operated under Contract No. DE-AC05-76RL01830 (EMSL). The scattering portion of this work further acknowledges the support of the Genomic Science Program, under Contract FWP ERKP752 and the Center for Structural Molecular Biology (CSMB) under Contract FWP ERKP291, Office of Biological and Environmental Research, U.S. Department of Energy and the High Flux Isotope Reactor supported by the Basic Energy Sciences, U.S. Department of Energy. The LiX beamline is part of the Center for BioMolecular Structure (CBMS), which is primarily supported by the National Institutes of Health, National Institute of General Medical Sciences (NIGMS) through a P30 Grant (P30GM133893), and by the DOE Office of Biological and Environmental Research (KP1605010). LiX also received additional support from NIH Grant S10 OD012331. As part of NSLS-II, a national user facility at Brookhaven National Laboratory, work performed at the CBMS is supported in part by the U.S. Department of Energy, Office of Science, Office of Basic Energy Sciences Program under contract number DE-SC0012704. This manuscript has been coauthored by UT-Battelle, LLC, under Contract No. DE-AC05- 00OR22725 with the U.S. Department of Energy. The United States Government retains and the publisher, by accepting the article for publication, acknowledges that the United States Government retains a non-exclusive, paid-up, irrevocable, worldwide license to publish or reproduce the published form of this manuscript or allow others to do so, for United States Government purposes.

### CONTRIBUTIONS

DD, HMO and ERM, together with ZPS, FV and SVP conceived the research plan and designed the experiments. DD produced the proteins in *Pichia pastoris* and ZPS together with PS prepared the holocellulose samples. MS, SVP and HMO conducted the SANS experiments and together with DD, ZPS, ERM and FV, interpreted the results. WL constructed the *Pca*LOOL model structures and together with and JB performed the SEC-SAXS analyses. GWB produced *Pca*LOOL12 in *Escherichia coli* and solved the *Pca*LOO12 NMR solution structure. DD, HMO and ERM drafted the manuscript with major contributions from all co-authors. All authors reviewed and finalized the manuscript.

### ETHICS DECLARATION

**Ethics approval and consent to participate.**

Not applicable.

### CONSENT FOR PUBLICATION

Not applicable.

### COMPETING INTERESTS

The authors certify that there is no competing of interest.

## Supplementary Files

This is a list of supplementary files associated with this preprint. Click to download.

- DahiyaSupplMaterialssubmitted18July2024.pdf
